# Supplementary material for: Content-rich biological network constructed by mining PubMed abstracts
Source: BMC Bioinformatics. 2004 Oct 8;5:147. doi: 10.1186/1471-2105-5-147 (PMC528731; doi:10.1186/1471-2105-5-147)
Supplement: Additional File 2 — The original results of the above study (non-essential files are deleted to keep the file size under the limit set by BMC bioinformatics). [file 1471-2105-5-147-S2.bz2 › chilibotAdditionalFile2/dip40/766ID9732274E18155/html/ENO_PNP.html]

 


 **ENO** and **PNP** 
  
Found 93 abstracts in PubMed, retrieved 40.  
 

 What does Google say? 
 PDF only 
| .edu only 

---

**Interactive relationship** (e.g. stimulation, inhibition, etc)

**Neutral relationship**- Earlier work has shown that the C terminal region of RNase E is a scaffold for the binding of degradosome components and has identified specific RNase E segments necessary for its interaction with polynucleotide phosphorylase  [ **PNP** ]  PNPase  [ **PNP** ] , RhlB RNA helicase, and enolase  [ **ENO** ] .  Ref: 11134527 Proc Natl Acad Sci U S A, 2001
- RNase E is a large multidomain protein with N terminal ribonucleolytic activity, an RNA binding domain and a C terminal scaffold that binds PNPase  [ **PNP** ] , enolase  [ **ENO** ]  and RhlB.  Ref: 12035760 Biochem Soc Trans, 2002
- We also show that the C terminal half of E. coli RNase E is both sufficient and necessary for its physical interaction with the 3 5 exoribonuclease polynucleotide phosphorylase  [ **PNP** ] , the RhlB helicase, and the glycolytic enzyme enolase  [ **ENO** ] , which are components of a degradosome complex.  Ref: 9751718 Proc Natl Acad Sci U S A, 1998
- Most of the tumor cells stained positively for chromogranin A and neuron specific enolase  [ **ENO** ]  NSE, and some of the tumor cells were positive for pancreatic polypeptide  [ **PNP** ] .  Ref: 10521965 Hepatogastroenterology< MedlineTA>Hepatogastroenterology, 1998
- All tumors also were immunoreactive for keratin, neuron specific enolase  [ **ENO** ] , and chromogranin, but only two patients had tumors that reacted for serotonin, pancreatic polypeptide  [ **PNP** ] , and vasoactive intestinal polypeptide.  Ref: 8402487 Cancer, 1993

**Non-interactive relationship** (e.g. studied together, co-existance, homology, etc.)

- However, binding of RhlB or PNPase  [ **PNP** ]  to enolase  [ **ENO** ]  was not detected under the same conditions.  Ref: 12181321 J Biol Chem, 2002
- No immunoreactive product was formed using antisera against somatostatin, 5 hydroxytryptamine, insulin, avian pancreatic polypeptide  [ **PNP** ] , motilin, cholecystokinin, secretin, neurotensin, vasoactive intestinal polypeptide, bombesin, neuron specific enolase  [ **ENO** ] , prochymosin, and pepsinogen.  Ref: 8162283 Cell Transplant< MedlineTA>Cell Transplant, 2002
- In addition, these tumors were also immunopositive for neuron specific enolase  [ **ENO** ] , S 100 protein, neurofilament protein, pancreatic polypeptide  [ **PNP** ] , chromogranin A, somatostatin, leuenkephalin, substance P and vasoactive intestinal peptide, as has been described in previous reports.  Ref: 8823994 Fukushima J Med Sci, 1995
- Such beta cells strongly immunostained for insulin, pancreatic polypeptide  [ **PNP** ] , and neuron specific enolase  [ **ENO** ] .  Ref: 11464630 Rev Gastroenterol Mex< MedlineTA>Rev Gastroenterol Mex, 1995
- Immunohistochemically the tumor was found positive for somatostatin, neuron specific enolase  [ **ENO** ]  and pancreatic polypeptide  [ **PNP** ] .  Ref: 8066887 Ugeskr Laeger, 1994
- These were ornithine carbamoyltransferase, phosphoglycerate kinase, nonphosphorylating glyceraldehyde 3 phosphate dehydrogenase, purine nucleoside phosphorylase  [ **PNP** ] , enolase  [ **ENO** ] , and glucose 6 phosphate isomerase.  Ref: 11854208 Infect Immun, 2002
- Nine human exocrine pancreatic adenocarcinomas were examined by serial sectioning and double and triple labeled immunohistochemical techniques with antibodies against chromogranin A, insulin, islet amyloid polypeptide, glucagon, somatostatin, pancreatic polypeptide  [ **PNP** ] , serotonin, pancreastatin, and neuron specific enolase  [ **ENO** ] .  Ref: 8379530 Am J Clin Pathol, 1993
- The tumour was diagnosed histopathologically as a moderately differentiated adenocarcinoma with focal neuroendocrine cell differentiation and dispersed cells reacting with antisera against neurone specific enolase  [ **ENO** ] , S 100 protein, neuropeptide Y, follicle stimulating hormone, substance P, vasoactive polypeptide VIP, adrenocorticotropic hormone and pancreatic polypeptide  [ **PNP** ]  PP as well as to one of three tested antisera raised against antidiuretic hormone ADH.  Ref: 9566647 J Intern Med, 1998
- Therefore, general tumor markers such as chromogranin A, pancreatic polypeptide  [ **PNP** ] , serum neuron specific enolase  [ **ENO** ]  and subunits of glycoprotein hormones have been used for screening purposes in patients without distinct clinical hormone related symptoms.  Ref: 10940685 Digestion, 2000
- PROCEDURE Immunohistochemical analysis was performed on the 42 tissue specimens, using antisera against CgA, neuron specific enolase  [ **ENO** ] , insulin, somatostatin, glucagon, and pancreatic polypeptide  [ **PNP** ] .  Ref: 9185968 Am J Vet Res, 1997
- Twenty two pancreatic islet cell tumors and normal pancreatic islets from ferrets were evaluated by immunohistochemistry for expression of the peptide hormones insulin, somatostatin, glucagon, and pancreatic polypeptide  [ **PNP** ]  PP and the neuroendocrine markers chromogranin A CgA and neuron specific enolase  [ **ENO** ]  NSE.  Ref: 9381649 Vet Pathol, 1997
- Therefore, general markers such as chromogranin A, pancreatic polypeptide  [ **PNP** ] , serum neuronspecific enolase  [ **ENO** ]  and subunit of glycoprotein hormones have been used for screening purposes in patients without distinct clinical hormone related syndromes.  Ref: 10604122 Ital J Gastroenterol Hepatol, 1999
- Seventeen rectal neuroendocrine tumors Rectal Carcinoids were studied by immunohistochemistry using antibodies directed against neuroendocrine markers chromogranin A, neuron specific enolase  [ **ENO** ] , synaptophysin, neuroendocrine peptides ACTH, glicentin, glucagon, pancreatic polypeptide  [ **PNP** ] , somatostatin, vasoactive intestinal peptide and antibody against serotonin.  Ref: 8767675 Ann Pathol, 1996
- Using an E. coli two hybrid system, together with BIAcore apparatus, we investigated the ability of three proteins, polynucleotide phosphorylase  [ **PNP** ]  PNPase  [ **PNP** ] , RhlB RNA helicase, and enolase  [ **ENO** ] , a glycolytic protein, to interact physically and functionally independently of RNase E.  Ref: 12181321 J Biol Chem, 2002
- An immunohistochemical study was performed on deparaffinized sections using the following antibodies neuron specific enolase  [ **ENO** ] , chromogranin A or synaptophysin, insulin, glucagon, somatostatin, pancreatic polypeptide  [ **PNP** ] , vasoactive intestinal peptide, gastrin, adrenocorticotropic hormone, alpha subunit of human chorionic gonadotropin, gonadotropin releasing factor, serotonin, and calcitonin.  Ref: 8898842 Am J Surg Pathol, 1996
- The Escherichia coli degradosome is a multienzyme complex with four major protein components the endoribonuclease RNase E, the exoribonuclease PNPase  [ **PNP** ] , the RNA helicase RhlB and enolase  [ **ENO** ] .  Ref: 9383162 Mol Microbiol, 1997
- Immunocytochemical markers of neuroendocrine cells, i.e. neuron specific enolase  [ **ENO** ] , chromogranin and synaptophysin as well as basal hormones, i.e. insulin, glucagon, somatostatin, pancreatic polypeptide  [ **PNP** ]  and, additionally, serotonin and gastrin were detected in serial sections.  Ref: 7843463 Folia Histochem Cytobiol, 1994
- N terminal sequencing of these proteins revealed the presence in the RNase E complex not only of polynucleotide phosphorylase  [ **PNP** ]  but also of DnaK, RNA helicase, and enolase  [ **ENO** ] .  Ref: 8632981 Proc Natl Acad Sci U S A, 1996
- In Escherichia coli, the exoribonuclease polynucleotide phosphorylase  [ **PNP** ]  PNPase  [ **PNP** ] , the endoribonuclease RNase E, a DEAD RNA helicase and the glycolytic enzyme enolase  [ **ENO** ]  are associated with a high molecular weight complex, the degradosome.  Ref: 11680851 RNA, 2001
- This observation is consistent with the finding that in the presence of 0.5 mM AMP  **PNP** , a nonhydrolysable analogue of ATP, there is an increased association of enolase  [ **ENO** ]  with microtubules.  Ref: 10727018 Eur J Cell Biol, 2000
- The development and histopathology of the pancreatic tumors were studied by light microscopy and immunocytochemistry for large T antigen, neuron specific enolase  [ **ENO** ] , insulin, proinsulin, glucagon, somatostatin, pancreatic polypeptide  [ **PNP** ] , gastrin, and serotonin.  Ref: 8388173 Am J Pathol, 1993
- Immunohistochemical analysis revealed staining for chromogranin A, neuron specific enolase  [ **ENO** ] , Leu 7, and synaptophysin, as well as pancreatic polypeptide  [ **PNP** ] .  Ref: 7688215 Arch Pathol Lab Med, 1993
- Whereas PNPase  [ **PNP** ]  and enolase  [ **ENO** ]  are present in E. coli in large excess relative to RNase E and therefore are detected in cells largely as molecules unlinked to the RNase E scaffold, immunogold labeling and biochemical analyses show that helicase is present in approximately equimolar amounts to RNase E at all cell growth stages.  Ref: 11134527 Proc Natl Acad Sci U S A, 2001
- Immunohistochemistry revealed predominant expression of motilin containing cells, with rare cells expressing somatostatin and pancreatic polypeptide  [ **PNP** ] , and staining for only one panendocrine marker, neurone specific enolase  [ **ENO** ] .  Ref: 11842283 Digestion, 2001
- Our results demonstrate that the carboxy terminal half CTH of ribonuclease E RNase E contains the binding sites for the three other major degradosomal components, the DEAD box RNA helicase RhlB, enolase  [ **ENO** ] , and polynucleotide phosphorylase  [ **PNP** ]  PNPase  [ **PNP** ] .  Ref: 9732274 Genes Dev, 1998
